# Nervonic Acid from *Malania oleifera* Reverses Parkinson’s Disease by Regulating Oxidative Stress, Neuroinflammation, and Gut Microbiota

**DOI:** 10.34133/bmr.0349

**Published:** 2026-05-08

**Authors:** Yuwen Li, Xiao Peng, Yang Xu, Jingyu Zhao, Yong Chen, Xuesong Liu, Lihua Peng

**Affiliations:** ^1^ College of Pharmaceutical Sciences, Zhejiang University, Hangzhou 310058, PR China.; ^2^ Jinhua Institute of Zhejiang University, Jinhua 321299, Zhejiang, PR China.

## Abstract

Parkinson’s disease (PD) is a neurodegenerative disease characterized by the progressive loss of dopaminergic neurons in the substantia nigra, accompanied by oxidative stress and neuroinflammation. While nervonic acid (NA) is recognized as an essential component of myelin sphingolipids, its specific therapeutic mechanisms in PD have not been thoroughly elucidated. In this study, we investigated the neuroprotective potential of NA derived from *Malania oleifera* and elucidated its underlying multimechanistic effect. We demonstrated that NA targets the core drivers of neuronal injury by restoring PTEN-induced kinase 1–Parkin-mediated mitophagy to alleviate mitochondrial dysfunction and oxidative stress. Concurrently, NA reprograms microglia from the pro-inflammatory M1 to the anti-inflammatory M2 phenotype, thereby inhibiting cytokine secretion and resolving neuroinflammation. Beyond these central protective effects, metabolomics analysis revealed that NA serves as a direct biosynthetic precursor for long-chain ceramides, facilitating myelin regeneration. In 1-methyl-4-phenyl-1,2,3,6-tetrahydropyridine-induced PD mice, these synergistic mechanisms collectively preserved dopaminergic neurons and improved motor function substantially. Furthermore, 16S ribosomal RNA amplicon sequencing revealed an associative remodeling of the gut microbiota, specifically enriching beneficial genera such as *Akkermansia*. Collectively, this study establishes NA as a potent multitarget therapeutic that concurrently regulates oxidative stress, neuroinflammation, and myelin regeneration to alleviate PD pathology.

## Introduction

Parkinson’s disease (PD) is the second most common neurodegenerative disease, affecting up to 2% of individuals over the age of 60 [1]. PD is characterized by the degeneration of dopaminergic (DAergic) neurons in the substantia nigra [2], accompanied by cognitive impairment and behavioral deficits. The primary pathological feature of PD is the aggregation of α-synuclein in DAergic neurons, which is further associated with inflammatory reaction and oxidative stress [3,4]. Although various therapeutic strategies, including dopamine activators, chemical molecules [5–7], and nucleic acids [8,9], have been extensively investigated [10], they are still seriously hindered by limited therapeutic efficiency and obvious side effects.

Other than these traditionally characterized pathologies, growing evidence has suggested that PD individuals also exhibit white matter lesions, including oligodendrocyte dysfunctions and myelination abnormalities [11–13]. These symptoms will, in turn, aggravate the pathology in the substantia nigra [11]. Therefore, developing novel therapeutic methods to inhibit oxidative stress and neuroinflammation and promote myelin regeneration presents a promising approach for PD treatment.

Nervonic acid (NA) is a important component of the very long-chain fatty acid family [14], mainly contained in the brain in the form of sphingolipids and has been identified with critical biological functions in myelin development and neural repair [15]. Perturbations of NA and its derivatives in the brain are associated with many neurological disorders, including multiple sclerosis [15], Alzheimer’s disease, and PD [16,17]. However, NA cannot be synthesized in sufficient quantities by humans and must be obtained from the diet. While present in sources like breast milk, the level of sphingolipids containing NA is markedly affected by aging, thus leading to a range of neurodegenerative diseases [18]. It is therefore hypothesized that NA supplementation might be an alternative strategy for PD symptom remission by endogenous therapeutics with minimal side effects induced.

Although previous studies have confirmed the potential of NA in alleviating PD motor symptoms and reducing general oxidative stress and inflammatory responses [17,19], the specific upstream mechanisms responsible for these effects remain unclear. Crucially, whether NA exerts neuroprotection by regulating mitochondrial quality control or by immunomodulation has not been fully elucidated. Furthermore, clinical studies have demonstrated that PD patients often experience gastrointestinal symptoms prior to motor symptoms [20], accompanied by marked gut microbiota dysbiosis [21]. Given that the gut microbiota can influence the central nervous system indirectly via immune pathways and microbiota-derived metabolites [22,23] and considering that oral administration is the standard route for NA, prior studies have largely overlooked the interaction between dietary NA and the gut microbiota, leaving the peripheral mechanisms of NA in PD unexplored. Therefore, it is hypothesized that NA may regulate gut microbiota.

Accordingly, herein, we provide a comprehensive investigation into the efficacy and potential mechanism of NA from *Malania oleifera* in oral administration for PD, with a particular focus on the interactions of NA with the gut microbiota, which have not been previously reported. It is expected to provide an important candidate drug molecule for neural repair and gut microbiota regulation in PD therapy.

## Materials and Methods

### Isolation and purification of NA

*M. oleifera* was purchased from Yunnan Beimu Biotechnology Co., LTD. *M. oleifera* oil was extracted using supercritical carbon dioxide extraction. Dried *M. oleifera* was crushed and placed in an extraction kettle. The optimized extraction conditions were as follows: extraction kettle pressure, 30 MPa; extraction temperature, 45 °C; separation kettle pressure, 10 MPa; desorption temperature, 55 °C; and extraction time, 2 h. Then, the oil was reflux-heated with 10% NaOH solution for 1 h. After the reaction, the mixture was cooled down and *M. oleifera* oil saponifiables were precipitated. Afterward, the saponifiables were purified using supercritical carbon dioxide extraction to remove unsaponifiables. The extraction conditions were as follows: extraction kettle pressure, 15 MPa; extraction temperature, 35 °C; and extraction time, 1 h. Then, the saponifiables were redissolved in 10% sulfuric acid and stirred in an 85 °C water bath for 2 h. To obtain NA, the precipitate was cooled, rinsed with distilled water until neutral, and then dried.

### HPLC–Q-TOF-MS/MS analysis

The obtained sample was identified by high performance liquid chromatography–quadrupole time of flight tandem mass spectrometry (HPLC–Q-TOF-MS/MS) analysis on a Sciex TripleTOF mass spectrometer (TripleTOF 5600+, AB Sciex, USA) equipped with an electrospray ionization source. The sample was separated by an ultra-high-performance liquid chromatography (UHPLC) system (Acquity Ultra, Waters, USA). The mobile phase was composed of A, 0.1% formic acid solution, and B, methanol (A:B = 10:90). The chromatographic conditions were as follows: injection volume, 3 μl; flow rate, 0.3 ml/min; and ultraviolet detector, 210 and 230 nm. The mass parameters were optimized as follows: ion source temperature, 550 °C; nebulization gas, 55 psi; curtain gas, 35 psi; spray voltage, 5,500 V in positive ion mode and −4,500 V in negative ion mode; declustering potential, 80 V; and collision energy, 10 V. Mass spectrometry (MS) and MS/MS scanning ranged from *m*/*z* 100 to 800 and 50 to 500, respectively. To obtain comprehensive structural information, the sample was analyzed in both positive and negative modes. The collected data and analysis were processed using SCIEX OS 3.3.1 (AB Sciex, USA).

### X-ray diffraction

The NA crystals were characterized using an x-ray diffraction (XRD) spectrophotometer (Ultima IV, RIKEN, Japan). The detection parameters were as follows: tube pressure, 40 kV; tube current, 40 mA; scan range, 5° to 80°; scan rate, 10° min^−1^; and sweep step, 0.02°.

### Cell culture

The SH-SY5Y cell line and BV2 cell line were acquired from the Shanghai Institute of Biological Science, Chinese Academy of Sciences. Cells were maintained in Dulbecco’s modified Eagle’s medium supplemented with 10% fetal bovine serum and 1% penicillin–streptomycin solution (Gibco BRL, USA). Cultures were incubated at 37 °C in a humidified 5% CO_2_ atmosphere, with media refreshed every 2 d.

### Cell viability assay

Cell viability was analyzed using Cell Counting Kit-8 (CCK-8) (AR1160-500, Boster, China) following the manufacturer’s instructions. Briefly, SH-SY5Y cells were seeded into 96-well plates at 5 × 10^3^ cells/well and allowed to adhere overnight. Cells were then treated with NA dissolved in dimethyl sulfoxide in 200 μl of fresh complete medium. After 24-h incubation, cells were gently washed with phosphate-buffered saline (PBS) and incubated with 100 μl of medium containing 10% CCK-8 reagent for 1 h at 37 °C. Finally, the optical density value was measured using a Multiskan FC microplate reader (Thermo Fisher Scientific, USA) at 450 nm, with cell viability expressed as a percentage relative to untreated controls.

### ROS assay

Intracellular reactive oxygen species (ROS) levels were analyzed using the Reactive Oxygen Species Assay Kit (S0033, Beyotime, China) according to the manufacturer’s instructions. Briefly, SH-SY5Y cells were seeded into 12-well plates at 8 × 10^4^ cells/well and allowed to adhere overnight. Cells were then treated with NA (NA-L, 50 μM, or NA-H, 100 μM) in 200 μl of fresh complete medium. After 24-h incubation, cells were gently washed with PBS, trypsinized, and centrifuged at 1,200 rpm for 5 min. The precipitate was resuspended in diluted 2′,7′-dichlorodihydrofluorescein diacetate and incubated at 37 °C for 60 min in the dark. After PBS washing, cells were transferred to a black 96-well plate with a clear bottom. Finally, fluorescence intensity was measured using a Multiskan FC microplate reader (Thermo Fisher Scientific, USA) at 488-nm excitation/525-nm emission, with ROS levels expressed as a percentage relative to untreated controls.

### MDA and GSH assay

The levels of malondialdehyde (MDA) and glutathione (GSH) in cells were analyzed using the Lipid Peroxidation MDA Assay Kit (S0131S, Beyotime, China) and a GSH detection kit (S0053, Beyotime, China) according to the manufacturer’s instructions. Briefly, SH-SY5Y cells were seeded into 6-well plates at 5 × 10^6^ cells/well and allowed to adhere overnight. Cells were then treated with NA (NA-L, 50 μM, or NA-H, 100 μM) in 200 μl of fresh complete medium. After 24-h incubation, cells were collected for analysis. The absorbance was measured using a Multiskan FC microplate reader (Thermo Fisher Scientific, USA), and the concentrations were normalized to the total protein content.

### RNA extraction and transcriptome analysis

The total RNA of SH-SY5Y cells was extracted using TRIzol (Thermo Fisher, USA) following the manufacturer’s instructions, with an additional DNase treatment. The concentration and purity of the RNA were assessed using NanoDrop (Thermo Fisher, USA), and the integrity of RNA was assessed using Bioanalyzer 2100 (Agilent, USA). For library preparation, messenger RNA was enriched from total RNA using poly-T oligo-attached magnetic beads. After fragmentation, complementary DNA synthesis was performed via reverse transcription with random hexamers, followed by second-strand synthesis. Quality-controlled libraries (Qubit quantification and bioanalyzer size distribution) were sequenced on an Illumina NovaSeq platform to generate 150-bp paired-end reads. Raw reads in fastq format were first processed through the fastp software to perform quality control, during which adapter sequences, reads containing poly-N, and low-quality reads were filtered out. The resulting high-quality reads were then aligned to the reference genome using HISAT2. Transcript assembly was subsequently performed using StringTie, followed by gene-level read counting with featureCounts. Gene expression levels were quantified as fragments per kilobase of transcript per million mapped reads (FPKM) values, which normalize read counts by both gene length and sequencing depth. Differentially expressed genes (DEGs) were identified using a threshold of |log_2_(fold change)| ≥ 0.58 (1.5-fold change) with *P* values ≤ 0.05. These significant gene sets were then subjected to functional enrichment analysis using the clusterProfiler R package, which incorporated gene length bias correction. Gene Ontology (GO) enrichment analysis (http://geneontology.org/) and Kyoto Encyclopedia of Genes and Genomes enrichment analysis (http://www.genome.jp/kegg/) were performed to elucidate the biological functions and pathways associated with the DEGs.

### Untargeted metabolome analysis

Cells were resuspended with prechilled 80% methanol and thoroughly homogenized by vortex mixing, followed by incubation on ice. After sonification for 6 min, the samples were centrifuged at 5,000 rpm for 1 min at 4 °C. The supernatant was lyophilized and subsequently dissolved in 10% methanol for liquid chromatography (LC)–MS/MS analysis. Chromatographic separation was achieved using a Vanquish UHPLC system (Thermo Fisher, USA) equipped with a Hypersil GOLD C18 column (100 × 2.1 mm, 1.9 μm). The mobile phase consisted of (A) 0.1% formic acid in water and (B) methanol, delivered at a flow rate of 0.2 ml/min with the following gradient program: 0 to 1.5 min, B maintained at 2%; 1.5 to 3 min, B goes from 2% linear change to 85%; 3 to 10 min, B goes from 85% linear change to 100%; 10 to 10.1 min, B goes from 100% linear change to 2%; 10.1 to 12 min, B maintained at 2%; 9 to 9.1 min, B goes from 40% linear change to 95%; and 9.1 to 12 min, B maintained at 95%. Mass spectrometric detection was performed using an Orbitrap Q Exactive HF-X mass spectrometer (Thermo Fisher, USA) operating in positive/negative mode with the following optimized parameters: spray voltage, 3.5 kV; capillary temperature, 320 °C; sheath gas, 35 psi; aux gas, 10 l/min; S-lens RF level, 60; and auxiliary gas heater temperature, 350 °C.

### Phenotypic transformation and identification of BV2 cells

BV2 cells were seeded into 12-well plates at a density of 10^5^ cells/well and allowed to adhere overnight. Cells were then treated with culture media, lipopolysaccharide (LPS; 1 μg/ml), or NA (50 or 100 μM) together with LPS (1 μg/ml), respectively. After 24 h, the cells were collected by trypsin digestion and labeled with fluorescein isothiocyanate (FITC)-conjugated anti-mouse CD86 antibody (105005, BioLegend, USA) and allophycocyanin-conjugated anti-mouse CD206 antibody (141707, BioLegend, USA) according to the manufacturer’s instructions. Flow cytometric immunofluorescence analysis was carried out using a flow cytometer (CytoFLEX S, USA).

### Assay of inflammatory cytokines

The inflammatory cytokines tumor necrosis factor-α (TNF-α), interleukin-6 (IL-6), and interleukin-1β (IL-1β) were analyzed using an enzyme-linked immunosorbent assay (ELISA) kit (EK282EG/EK206/EK201BEGA, MULTI SCIENCE, China). Briefly, BV2 cells were seeded into 12-well plates at a density of 10^5^ cells/well and allowed to adhere overnight. Cells were then treated with culture media, LPS (1 μg /ml), or NA (50 or 100 μM) together with LPS (1 μg /ml), respectively. Media were subsequently collected for ELISAs. The specific processes were performed according to the manufacturer’s protocols.

### Animal treatment

Six-week-old C57BL/6 mice (Shanghai Slack Laboratory Animal Co. Ltd) were acclimatized for 1 week before experimentation under standard housing conditions (25 ± 1 °C, 12-h light/dark cycle). All experimental procedures were approved by the Institutional Animal Care and Use Committee of Zhejiang University (ZJU20170733). The mice were randomly divided into 4 groups (*n* = 6), control, model, NA-L (40 mg/kg), and NA-H (80 mg/kg), with 6 mice in each group. To establish the PD model, mice except for the blank group were intraperitoneally injected with MPTP at a dose of 30 mg/kg for 7 d. The NA treatment steps are as follows: (a) Apply 100 μl of corn oil in the blank and model groups. (b) Apply 100 μl of NA corn oil solution (8 mg/ml) to the NA-L group. (c) Apply 100 μl of NA corn oil solution (16 mg/ml) to the NA-H group. The animals received the drug via gavage every 2 d for a duration of 14 d. After sacrifice, the tissues were collected and allocated for different assays to maximize data acquisition. Randomly selected 3 brains were utilized for Western blot (WB), and the remaining 3 brains were perfused for histological analysis (*n* = 3). All analyses were performed in a blinded manner.

### Fatty acid extraction and measurement

The brain tissue samples were weighed accurately and homogenized in ice-cold 0.9% sodium chloride solution. Total lipids were extracted using a modified Folch method. Briefly, an aliquot of tissue homogenate was mixed with a chloroform/methanol mixture (2:1, v/v). The mixture was vortexed vigorously for 30 s and then sonicated for 10 min to ensure complete lipid extraction. After centrifugation at 12,000 g for 10 min at 4 °C, the lower organic phase (chloroform layer) containing the lipids was carefully collected and evaporated to dryness under a gentle stream of nitrogen. The residue was redissolved in methanol and centrifuged to remove any particulates, and the supernatant was used for LC–MS/MS analysis. The quantitative analysis was performed on a Thermo Scientific Quantis Triple Quadrupole mass spectrometer (Thermo Fisher Scientific, USA) equipped with an electrospray ionization source. The sample separation was achieved using a Waters ACQUITY UPLC C18 column (2.1 mm × 50 mm, 1.7 μm). The mobile phase was composed of A, 0.1% formic acid solution, and B, acetonitrile (A:B = 5:95). The chromatographic conditions were as follows: flow rate, 0.3 ml/min; column temperature, 40 °C; and injection volume, 5 μl. The mass parameters were optimized as follows: spray voltage, 2.5 kV in negative ion mode; ion transfer tube temperature, 380 °C; auxiliary gas heating temperature, 380 °C; sheath gas, 15 Arb; and auxiliary gas, 50 Arb. Quantification was conducted in selected ion monitoring mode. The monitoring ions were set at *m*/*z* 365.2 (RF lens, 134 V) for NA. The collected data and analysis were processed using the Thermo Xcalibur software (Thermo Fisher Scientific, USA).

### Rotarod test

The behavioral assessments were performed in a quiet, temperature-controlled room. Animals were allowed to acclimate to the testing environment for 1 h prior to assessment. To eliminate observer bias, all behavioral experiments were conducted in a blinded manner. Before experimental testing, all mice underwent a standardized training protocol on an accelerating rotarod apparatus for 3 consecutive days. During training sessions, the rotation speed gradually increased from 0 to 40 rpm over 300 s. Animals that fell from the rotating rod were immediately returned to the apparatus and permitted a 60-s recovery period before resuming training. For formal experiments, the rotarod was programmed to automatically stop upon detection of a mouse falling onto the platform below. The duration that each mouse maintained on the rod, the maximum achieved rotation speed, and the total travel distance were recorded. The final data point for each animal represents the average of 3 consecutive successful trials.

### Hematoxylin and eosin staining

After dewaxing, the sections were washed and stained for 5 min using hematoxylin (Wuhan BiochiDu Biotechnology Co., Ltd, China). Then, the sections were immersed in 1% hydrochloric acid solution for 2 s to remove excess stain, immediately followed by a 30-s differentiation in ammonia water solution. After washing, the sections were dehydrated with 95% ethanol and stained with eosin solution for 5 to 8 s. After dehydration, the slides were mounted, and images were collected under a microscope. For quantitative analysis, the region of interest was strictly selected in the substantia nigra and cells with a clearly visible nucleus and intact cell body within the region of interest were manually counted.

### Nissl staining

After dewaxing, the sections were washed and stained for 5 min using Nissl stain (Wuhan Servicebio Biotechnology Co., Ltd, China) and then washed 3 times with distilled water. After dehydration, the slides were mounted, and images were collected under a microscope and quantified using the ImageJ software. A uniform threshold was set based on the background signal of the blank group and applied consistently across all images to calculate the area fraction (%).

### TH staining

After dewaxing, the sections were subjected to antigen repair by immersion in citric acid antigen repair solution (HKI0001, Wuhan BiochiDu Biotechnology Co., Ltd, China). Afterward, the sections were rinsed 3 times with PBS for 5 min each. Then, the sections were treated with 3% hydrogen peroxide and incubated at room temperature for 25 min, followed by three 5-min washes. Next, the sections were treated with 3% bovine serum albumin (BSA; Wuhan BiochiDu Biotechnology Co., Ltd, China) and incubated for 30 min at room temperature. After gently removing the blocking solution, the primary antibody against tyrosine hydroxylase (TH; 1:1,000, 25859-1-ap, Wuhan Mitaka Biotechnology Co., Ltd, China) was applied, and the sections were incubated overnight at 4 °C. After washing and drying, the sections were incubated with horseradish peroxidase (HRP)-labeled rabbit and mouse universal secondary antibody (I20012C, Tuling Hangzhou Biopharmaceutical Co., Ltd, China) at room temperature. After washing, chromogenic detection was performed using 3,3′-diaminobenzidine substrate solution. Upon achieving optimal staining intensity, the color development was terminated, followed by nuclear staining with hematoxylin (HK1024, Hangzhou Hulk Biotechnology Co., Ltd, China). After dehydration, the slides were mounted, and images were collected under a microscope and quantified using the ImageJ software. A uniform threshold was set based on the background signal of the blank group and applied consistently across all images to calculate the area fraction (%).

### Immunofluorescence staining

After dewaxing, the sections were immersed in 3% H_2_O_2_ methanol solution for 10 min to inhibit endogenous peroxidase. After washing with distilled water, the sections underwent incubation in 0.01 M citrate buffer. Then, the sections were washed 3 times with PBS and blocked with 5% BSA for 20 min at room temperature. The samples were subsequently incubated with FITC-conjugated secondary antibodies for 2 h, after which nuclear staining was performed using 4′,6-diamidino-2-phenylindole solution (3 μg/ml). For immunofluorescence staining, sections were incubated with the following primary antibodies: neuronal nuclei (NeuN; 1:300, M11954-3, Boster), nestin (1:200, ab182981, ABclonal), arginase 1 (ARG1; 1:200, GB11285, Servicebio), inducible nitric oxide synthase (iNOS; 1:200, 18985-1-AP, Proteintech Group), myelin basic protein (MBP; 1:500, BA0094, Boster), and HRP-labeled anti-mouse secondary antibodies (1:400, 5220-0341, SeraCare). Fluorescence visualization was conducted using a fluorescence microscope (Olympus BX61) with image acquisition performed through the Olympus Soft Imaging Solution software. All images were postprocessed and quantified using the ImageJ software. For quantification, a uniform threshold was set based on the background signal of the blank group and applied consistently across all images to calculate the integrated density (AU).

### WB analysis

Protein extraction was performed using radioimmunoprecipitation assay lysis buffer containing protease inhibitor cocktail (Thermo Fisher, 89900), with protein concentration determined by bicinchoninic acid assay (Beyotime, P0010). Sodium dodecyl sulfate–polyacrylamide gel electrophoresis gels were prepared with 5% stacking gel and 8% to 12% separating gel. Then, samples containing 60 μg of total protein (10 to 15 μl) were loaded per well and electrophoresed initially at 60 V through the stacking gel, followed by 80 V for separation for 2 h. For protein transmembrane, a polyvinylidene fluoride membrane (Millipore, IPVH00010) was immersed in methanol for 20 s and equilibrated in Tris–glycine transfer buffer (containing 5% methanol) for at least 5 min. Similarly, gels were equilibrated in transfer buffer for 30 min under cooling conditions before transfer at 100 V for 2 h. Membranes were subsequently blocked with 5% BSA in tris-buffered saline with Tween 20 (T-TBS) at room temperature for 1 h and washed with T-TBS for 5 min each. Primary antibody incubations were performed overnight at 4 °C using the following dilutions: glyceraldehyde-3-phosphate dehydrogenase (GAPDH; 1:10,000, 60004-1-Ig, Proteintech), sequestosome 1 (SQSTM1/p62; 1:2,000, ab56416, Abcam), Parkin (1:2,000, A11172, ABclonal), glycogen synthase kinase 3β (GSK-3β; 1:2,000, A11731, ABclonal), TNF-α (1:1,000, BA0131, BOSTER), and α-synuclein (1:1,000, ab212184, Abcam). Following T-TBS washes (3 × 5 min), membranes were incubated with HRP-conjugated goat anti-rabbit immunoglobulin G (IgG) (1:50,000, 5220-0336, SeraCare) and goat anti-mouse IgG (1:50,000, 5220-0341, SeraCare) secondary antibodies for 1 h at room temperature. After extensive washing with T-TBS (5 × 5 min), an enhanced chemiluminescence (ECL) working solution was prepared with SuperSignal West Dura Extended Duration Substrate (34075, Thermo Fisher). The membrane was incubated with the ECL solution at room temperature for 1 min, after which the excess ECL reagents were removed, and the membrane was sealed with plastic wrap. Membranes were exposed to x-ray film for 5 to 10 min for development and fixation. The density quantification was conducted using the ImageJ 1.8.0 software (USA). Data are presented as the ratio of the optical density of the target protein band to the GAPDH band.

### 16S rRNA sequencing

Targeted amplification of 16S ribosomal RNA (rRNA), 18S rRNA, and internal transcribed spacer (ITS) genes from distinct regions (16SV4, 16SV3, 16SV3–V4, 16SV4–V5, 18SV4, 18SV9, ITS1, ITS2, and ArcV4) was performed using barcoded region-specific primers (e.g., 16SV4, 515F-806R; 8SV4, 528F-706R; and 18SV9, 1380F-1510R). Polymerase chain reaction (PCR) amplification was carried out in 15-μl reaction volumes containing Phusion High-Fidelity PCR Master Mix (New England Biolabs), 0.2 μM of each primer, and 10 ng of template DNA. Thermal cycling conditions comprised the following: initial denaturation (98 °C, 1 min), 30 cycles of denaturation (98 °C, 10 s), annealing (50 °C, 30 s), elongation (72 °C, 30 s), and extension (72 °C, 5 min). PCR products were visualized via 2% agarose gel electrophoresis after mixing with SYBR Green-containing loading buffer. Equimolar pools of amplified products were created and purified before library preparation. Sequencing libraries were constructed with appropriate index adapters, quantified using Qubit fluorometry and quantitative real-time polymerase chain reaction, and size-verified by bioanalyzer electrophoresis. Pooled libraries were sequenced on Illumina platforms according to optimal cluster density and required sequencing depth specifications.

### Statistical analysis

Statistical analyses were performed with GraphPad Prism 10.1 (GraphPad Software, USA). All data are presented as mean ± SD. The Student *t* test was applied for comparisons between 2 groups, and one-way analysis of variance (ANOVA) with Tukey’s multiple comparisons test for multiple comparisons. A value of *P* < 0.05 was considered a significant difference. *P < 0.05, **P < 0.01, ***P < 0.001, and ****P < 0.0001. The ImageJ software was used for the quantification of images.

## Results

### The structural characterization of NA from *M. oleifera*

**Fig. 1.**
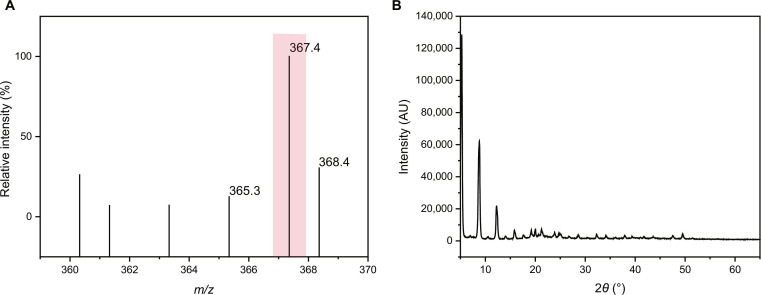
Structural characterization of nervonic acid (NA). (A) Mass spectrometry (MS) spectra of NA. (B) X-ray diffraction pattern of NA.

To obtain NA, dried *M. oleifera* kernels were utilized for extraction. The crude oil was obtained by supercritical carbon dioxide extraction, with an oil yield of approximately 32% (w/w). The content of NA in the crude oil was determined to be approximately 50%. Subsequently, the crude oil underwent purification processes to obtain NA crystals. The final purification yield of NA was 31%, which was calculated based on the mass of crude oil. The purity of the obtained NA was confirmed to be >99.99% by HPLC analysis (Fig. S1), ensuring its suitability for biological assays. For structural characterization, our previous study reported the results of ^1^H nuclear magnetic resonance, ^13^C nuclear magnetic resonance, and Fourier transform infrared spectroscopy analysis of NA that had been isolated from *M. oleifera* [24]. Subsequently, we performed HPLC–Q-TOF-MS and XRD analysis to further characterize the structure of NA. As shown in Fig. 1A, (M + 1) was characterized by the peak 367.3568, and the calculated molecular mass (366.4) matches the exact mass of NA (366.35). What is more, Fig. 1B displays the XRD pattern of NA, providing a novel approach for its identification.

### In vitro neuron-protective effect and mechanisms of NA

**Fig. 2.**
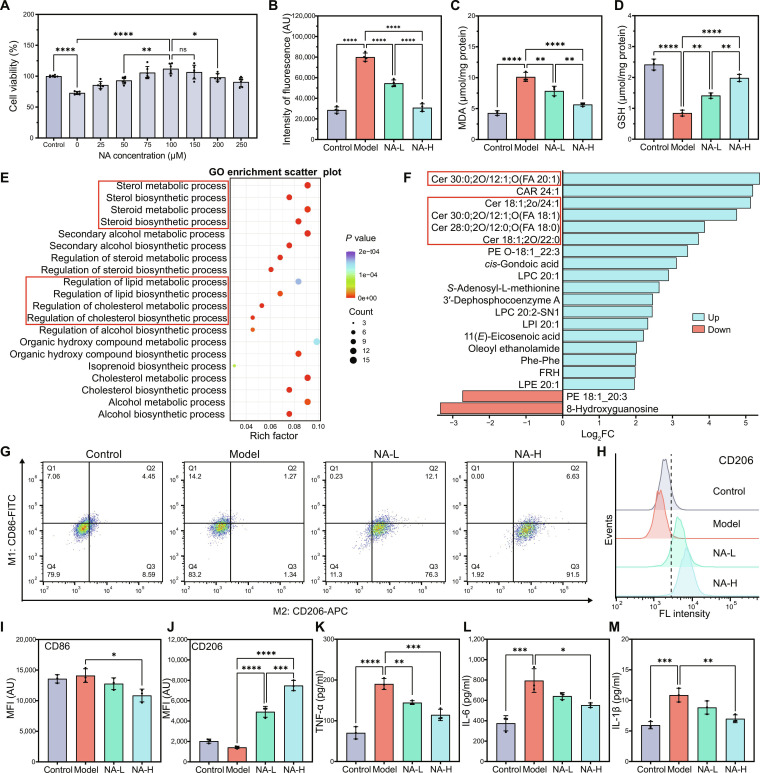
In vitro bioassays of nervonic acid (NA) and mechanistic investigation. (A) Cell viability (*n* = 6). (B) Oxidative stress (*n* = 4). (C) Malondialdehyde (MDA) assay. (D) Glutathione (GSH) assay. (E) Gene Ontology (GO) enrichment scatter plot of the differentially expressed genes (DEGs) (*P* < 0.05). (F) Differential metabolite matchstick map (top 20). (G) Representative flow cytometry image of BV2 microglia polarizing into M1 phenotype (CD86) or (H) M2 phenotype (CD206). (I) MFI of CD86. (J) Mean fluorescence intensity (MFI) of CD206. Expression of the proinflammation cytokines (K) tumor necrosis factor-α (TNF-α), (L) interleukin-6 (IL-6), and (M) interleukin-1β (IL-1β). ****P < 0.0001, ***P < 0.001, **P < 0.01, and *P < 0.05. Mean ± SD, *n* = 3.

The in vitro Parkinson’s disease model was established through administration of 1-methyl-4-phenylpyridinium ions (MPP^+^) [8]. To determine the optimal therapeutic concentration, the CCK-8 assay was performed. As shown in Fig. 2A, NA treatment markedly rescued the cell viability reduction induced by MPP^+^ in a dose-dependent manner. Since the 100 μM concentration yielded the maximal neuroprotective effect without inducing cytotoxicity, we designated 50 and 100 μM as the experimental doses for the subsequent studies. Further, we evaluated the protective effect of NA against oxidative stress. As shown in Fig. 2B, NA treatment down-regulated intracellular ROS levels markedly, with the ROS level decreasing by 66% to that of the model group. To further quantify the antioxidant capacity of NA, we measured the levels of MDA and GSH. As shown in Fig. 2C and D, the model group exhibited a drastic increase in MDA content and a marked depletion of GSH compared to the control group, indicating severe oxidative stress. However, NA treatment substantially inhibited the accumulation of MDA and restored intracellular GSH levels. Notably, the NA-H group demonstrated superior protective efficacy compared to the NA-L group.

To elucidate the mechanism underlying NA’s neural protective effects, transcriptomic analysis was then performed to analyze DEGs after NA treatment. As shown in Fig. S2, NA treatment up-regulated 91 DEGs and down-regulated 89 DEGs in total. Followingly, the GO enrichment analysis of the DEGs showed that the top significantly enriched biological processes were sterol, steroid, lipid, cholesterol metabolic, and biosynthesis processes (Fig. 2E). The results suggested that NA might be involved in membrane lipid synthesis, which was consistent with previous research findings [25,26].

Furthermore, we analyzed the differential accumulated metabolites between the model and NA groups. Notably, comparative analysis detected 150 differential accumulated metabolites exhibiting significant expression changes (Fig. S3). Among them, the levels of several ceramides, including Cer30:0;2O/12:1;O(FA20:1), Cer18:1;2O/24:1, Cer30:0;2O/12:1;O(FA18:0), Cer28:0;2O/12:0;O(FA16:0), and Cer18:1;2O/22:0, were significantly up-regulated (Fig. 2F). This up-regulation indicates that exogenously administered NA effectively refuels the sphingolipid metabolism. The results of metabolomics aligned with those of the GO analysis of DEGs. Ceramides are the simplest type of sphingolipid and the key molecules for sphingolipid synthesis. Within cells, ceramides are transported to the Golgi apparatus and serve as direct metabolic precursors for sphingomyelin and glycosphingolipid biosynthesis, providing the essential structural basis for maintaining membrane stability and medullary sheath integrity [18].

In the context of PD, a pathological imbalance characterized by excessive pro-inflammatory M1 state microglia activation with a reduced anti-inflammatory M2 response acts as a key driver of neuroinflammation and DAergic neurons degeneration [27]. To evaluate the immunomodulatory potential of NA, we assessed microglial polarization in BV2 cells via flow cytometry (Fig. 2G to J). Stimulation with LPS elicited a robust polarization toward the pro-inflammatory M1 phenotype, characterized by a marked surge in the population of CD86+ cells. Notably, NA treatment effectively reduced the M1 response and promoted a shift toward the anti-inflammatory M2 phenotype, as evidenced by the down-regulation of CD86 and the concurrent up-regulation of the M2 marker CD206 (Fig. 2G and H). Quantitatively, NA treatment reduced the mean fluorescence intensity of the M1 marker CD86 by approximately 23% (Fig. 2I) while remarkably boosting the mean fluorescence intensity of the M2 marker CD206 by 5.26-fold compared to that of the model group (Fig. 2J). Activated microglia normally produce pro-inflammatory cytokines such as TNF-α, IL-6, and IL-1β to promote neuroinflammation. Therefore, we assessed the secretion of cytokines in BV2 cells with different treatments using ELISA kits to confirm anti-inflammatory activity. The result showed that NA reduced the content of TNF-α, IL-6, and IL-1β by 39.9%, 30.3%, and 35.5%, respectively. These results provide direct evidence that NA mitigates neuroinflammation by reprogramming microglia from a pro-inflammatory M1 state to an anti-inflammatory M2 phenotype and reducing the release of inflammatory cytokines.

### Behavioral and histomorphological evaluation of mice upon NA intervention

**Fig. 3.**
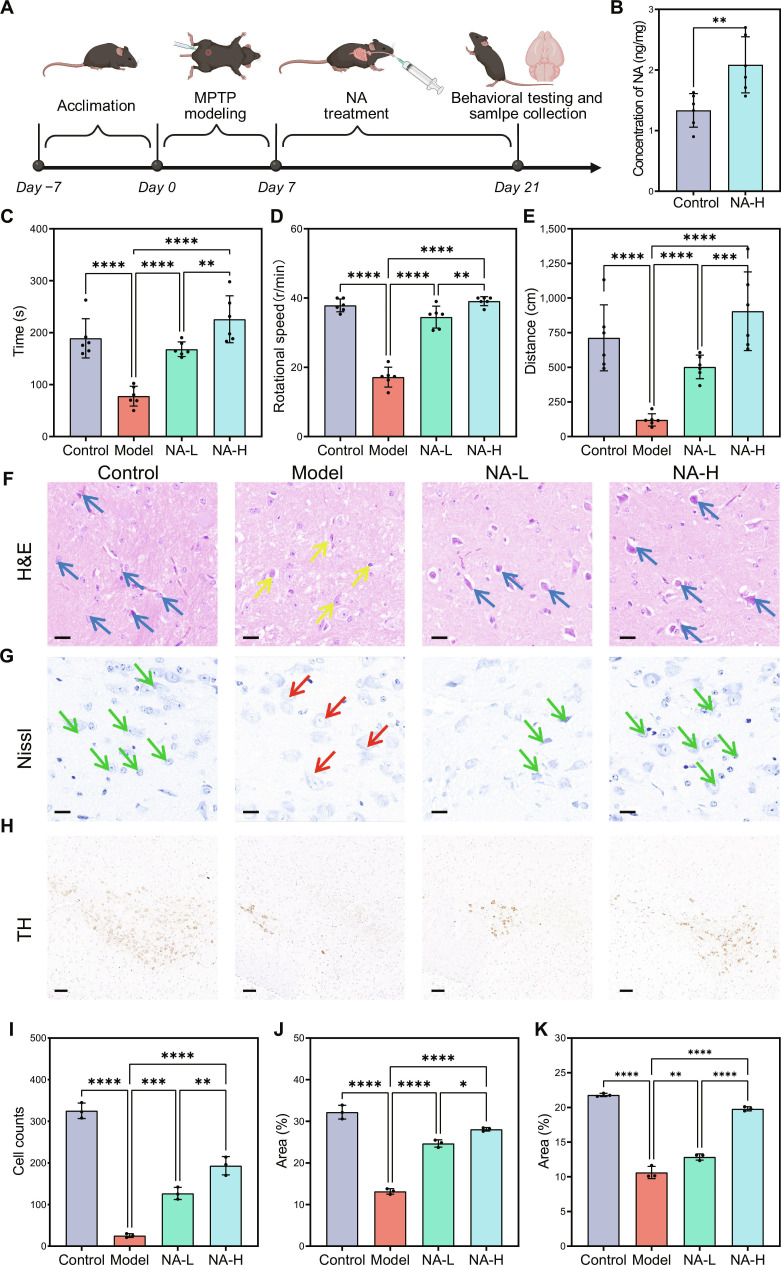
Behavioral and histomorphological evaluation of Parkinson’s disease (PD) mice. (A) Flowchart of the animal experiment process. (B) The concentration of nervonic acid (NA) in brain tissue. (C) Duration on the rod, (D) rotational speed, and (E) travel distance in the rotarod test (*n* = 6). (F) Hematoxylin and eosin (H&E) staining. Scalebar: 20 μm. (G) Nissl staining. Scalebar: 20 μm. (H) Tyrosine hydroxylase (TH) staining. Scalebar: 100 μm. (I to K) The quantitative analysis of (F) to (H), respectively (*n* = 3). ****P < 0.0001, ***P < 0.001, **P < 0.01, and *P < 0.05. Mean ± SD.

Building upon the promising in vitro findings demonstrating NA’s neuroprotective properties, we employed a combination of quantitative behavioral phenotyping with histomorphological analysis to systematically evaluate NA’s therapeutic efficacy in vivo (Fig. 3A). As shown in Fig. S4, there was no significant change in the body weight of the animals during NA administration. Firstly, to investigate whether orally administered NA could cross the blood–brain barrier and accumulate in the brain, we measure the total NA concentration in brain tissue after 14 d of every-other-day administration using HPLC–MS/MS. As shown in Fig. S5, the retention time of NA was stable at 4.55 min, and the calibration curve showed good linearity over the range of 10 to 100 ng/ml, ensuring the reliability of the quantification of NA (Fig. S6). As shown in Fig. 3B, the basal level of NA in the control group was 1.33 ± 0.27 ng/ml. The NA concentration in the treatment group was approximately 1.56-fold higher than that in the control group. This accumulation confirms that exogenous NA could effectively penetrate the blood–brain barrier and supplement the endogenous pool via oral administration.

Since PD is characterized by motor dysfunction, we evaluated the impact of NA on motor coordination using rotarod tests. Mice treated with NA demonstrated marked improvements in rotarod performance, exhibiting greater maximum speed tolerance, a longer endurance time, and an increased travel distance relative to the model group (Fig. 3C to E), suggesting NA’s therapeutic potential for motor dysfunction in the MPTP-induced PD model.

The characteristic degeneration of substantia DAergic neurons, a pathological hallmark of PD, was evaluated through hematoxylin and eosin staining, Nissl staining, and TH staining. Firstly, hematoxylin and eosin staining particularly revealed severe DAergic neuron loss in model group animals, manifested by disrupted neuronal distribution, nuclear condensation, and pathological cellular swelling (Fig. 3F, yellow arrows). Meanwhile, DAergic neurons under NA treatment exhibited improved appearance with typical morphology and reduced signs of degeneration (Fig. 3F, blue arrows). Particularly, quantitative analysis revealed a dose-dependent neuroprotection effect, with the NA-L and NA-H groups exhibiting 4.3-fold and 6.55-fold increases in DAergic neuron cell counts compared to the model group, respectively (Fig. 3I). Additionally, Nissl staining revealed that the model group showed a marked decrease in Nissl bodies and severe neuronal shrinkage (Fig. 3G, red arrows). In contrast, the NA treatment groups displayed substantial improvements in neuronal structure and numerous Nissl bodies (Fig. 3G, green arrows), suggesting that NA treatment enhanced the structural integrity of DAergic neurons. Specifically, the Nissl-positive area in the NA-L and NA-H groups increased substantially by 1.88-fold and 2.13-fold compared to that in the model group, respectively (Fig. 3J). Moreover, NA treatment exhibited markedly more effective protection against the decline of TH-positive DAergic neurons. Specifically, the NA-H group demonstrated superior DAergic neurons preservation (Fig. 3H), characterized by the greatest preservation of neuronal density and most intact TH-positive cell bodies and neural fibers, exhibiting a 1.96-fold increase in TH-positive area compared to the model group (Fig. 3K).

### Multidimensional neuroprotection of NA in PD mice

**Fig. 4.**
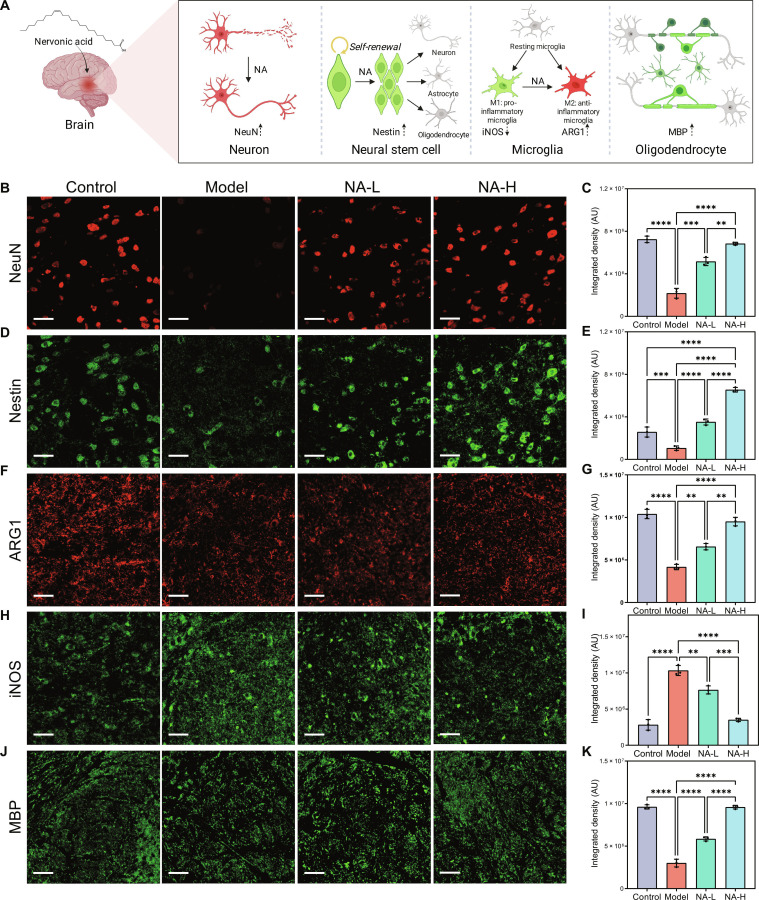
Neuroprotective effects of nervonic acid (NA) on multiple neuronal cell types (A). Immunofluorescence staining indicating the (B) neuronal nuclei (NeuN), (D) nestin, (F) arginase 1 (ARG1), (H) inducible nitric oxide synthase (iNOS), and (J) myelin basic protein (MBP) expression. Scalebar: 50 μm. (C, E, G, I, and K) The quantitative analysis of (B), (D), (F), (H), and (J), respectively. *n* = 3, ****P < 0.0001, ***P < 0.001, **P < 0.01, and *P < 0.05. Mean ± SD.

As an endogenous neuromodulator, NA demonstrates unique capabilities for axonal pathway reconstruction and neuronal regeneration. Therefore, we have further assessed the neuroprotective effects of NA on multiple neuronal cell types by immunofluorescence staining (Fig. 4A).

In PD progression, the reduced expression of NeuN in substantia nigra reflects global neuronal degeneration beyond the loss of DAergic neurons [28]. As shown in Fig. 4B, the red fluorescence of NeuN in the NA-H group was the most pronounced. Quantitative assessment of the positive expression integrated density aligns with the observations, where the NeuN expression of the NA-L and NA-H groups was 2.37-fold and 3.15-fold higher than that of the model group, respectively (Fig. 4C), suggesting the potential of NA in promoting neuronal regeneration.

Neural stem cells can differentiate into various neural cell types, aiding in the repair of damaged neural tissue, and they can also secrete neurotrophic factors to protect existing neural cells, reducing damage caused by oxidative stress [29]. As the marker of neural stem cells, the green fluorescence of nestin was enhanced in the NA-L and NA-H groups (Fig. 4D), with 3.32-fold and 6.18-fold higher counts, respectively (Fig. 4E). These results indicated that NA supplement can promote the proliferation and activation of neural stem cells and thus promote the repair and regeneration of nerve cells in the substantia nigra.

Microglia-mediated neuroinflammation has been described as another hallmark of PD in addition to the loss of DAergic neurons, and the mitigation of neuroinflammation relies on the activation of anti-inflammatory M2 microglia [30,31]. The substantia nigra tissue in the NA-L and NA-H groups reveals a substantial Arg-1 positive signal and a decreased iNOS positive signal (Fig. 4F and H), which are the markers of M2 and M1 phenotypes, respectively [32]. Particularly, the Arg-1 integrated density of the NA-H group is 2.28-fold higher than that of the model group, and the iNOS integrated density decreased by 26% and 66% after the treatment of NA-L and NA-H, respectively (Fig. 4G and I). These results indicated that NA could regulate microglial phenotypic shifts to relieve neuroinflammation.

The degeneration of the myelin sheath is one of the concomitant pathological features in the pathogenesis of PD [33]. Since oligodendrocytes are essential for myelin regeneration, we measured the fluorescence intensity of the MBP marker to assess oligodendrocyte activity [34]. As shown in Fig. 4J, the groups treated with NA both exhibited a marked increase in oligodendrocytes. Quantitative assessment aligns with the observations, where the MBP expression of the NA-L and NA-H groups was 1.95- and 3.19-fold higher than that of the model group, respectively (Fig. 4K), indicating that supplementation of NA can promote myelin regeneration and the recovery of damaged nerve fibers.

Collectively, the results of immunofluorescence showed that NA exerts neuroprotective effects through 2 major pathways. On one hand, NA modulates microglia phenotypes in the neuroinflammation of PD; on the other hand, NA promotes nerve regeneration in substantia nigra and myelination in white matter, exhibiting comprehensive protective effects against neurodegeneration in the brain.

### Molecular mechanism of NA protects DAergic neurons

**Fig. 5.**
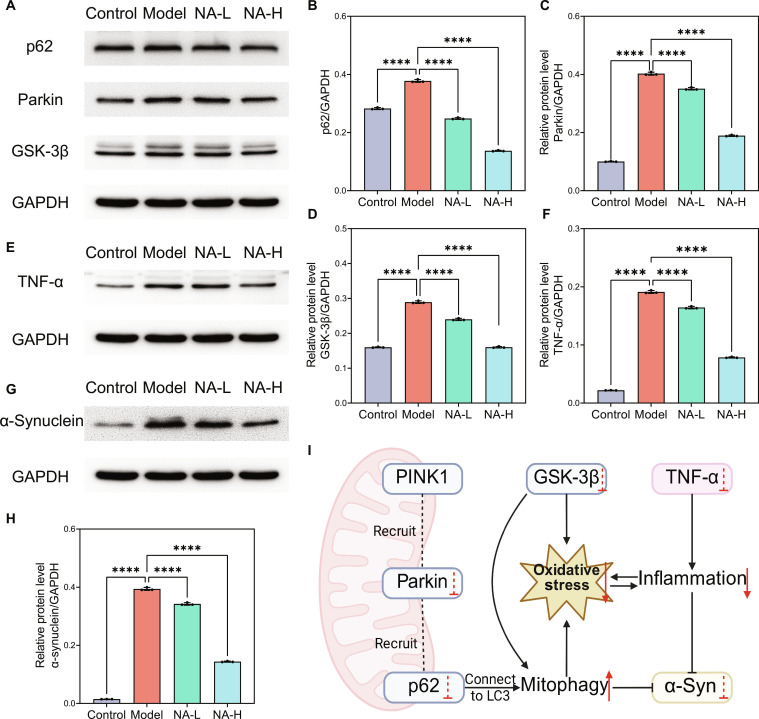
Molecular mechanism of nervonic acid (NA) protects dopaminergic (DAergic) neurons. (A, E, and G) Western blot (WB) analysis and (B to D, F, and H) quantitative results of sequestosome 1 (SQSTM1/p62), Parkin, glycogen synthase kinase 3β (GSK-3β), tumor necrosis factor-α (TNF-α), and α-synuclein levels (*n* = 3). (I) The schematic illustration of NA regulates PTEN-induced kinase 1 (PINK1)–Parkin-mediated mitophagy, relieves oxidative stress, and alleviates Parkinson’s disease (PD) neuropathology. ****P < 0.0001, ***P < 0.001, **P < 0.01, and *P < 0.05. Mean ± SD.

Mitochondrial dysfunction, inflammatory responses, and pathological α-synuclein accumulation are the 3 core pathological features of PD [3]. Mitochondrial dysfunction exacerbates the development of oxidative stress, which promotes the aggregation of α-synuclein [35]. To systematically investigate NA’s neuroprotective mechanisms targeting DAergic neuron survival, WB was employed to quantify its modulatory effects on these interrelated pathological markers. In the PTEN-induced kinase 1 (PINK1)–Parkin-mediated mitophagy, PINK1 accumulates on the outer membrane of damaged mitochondria due to the loss of membrane potential and then recruits and activates Parkin [36,37]. Subsequently, adaptor proteins, including SQSTM1/p62, accumulate on the outer mitochondrial membrane and then facilitate the recruitment of autophagosomes. These autophagosomes encapsulate the damaged mitochondria and deliver them to lysosomes for degradation, thereby helping maintain cellular homeostasis and prevent the accumulation of dysfunctional mitochondria [38]. Therefore, the expressions of SQSTM1/p62 and Parkin are negatively correlated with autophagic activity [8]. Moreover, accumulating evidence has demonstrated that the overactivation of GSK-3β exacerbates PD pathogenesis through the induction of mitochondrial dysfunction and oxidative stress [39]. The results showed that NA down-regulated the expression of SQSTM1/p62, Parkin, and GSK-3β by 63.7%, 53.0%, and 44.6% respectively (Fig. 5A to D), suggesting that NA treatment promotes mitophagy to alleviate oxidative stress against PD.

Furthermore, inflammation response is present throughout the entire progression of PD, and TNF-α, as a key pro-inflammatory factor, is often elevated in PD individuals. Inhibiting the action of TNF-α is considered a potential strategy for treating PD [40]. Figure 5E and F reveals that NA down-regulated the expression of TNF-α by 59.0%, indicating that NA treatment substantially relieves the inflammation in PD. Pathological α-synuclein accumulation is a hallmark of PD, which is closely related to the impaired mitophagy system [41]. As shown in Fig. 5G and H, the α-synuclein of the NA-H group decreased by 63.5% compared to that of the model group, indicating that NA alleviates the aggregation of α-synuclein through regulating PINK1–Parkin-mediated mitophagy and inflammatory response (Fig. 5I).

### Remodeling of gut microbiota through NA treatment

**Fig. 6.**
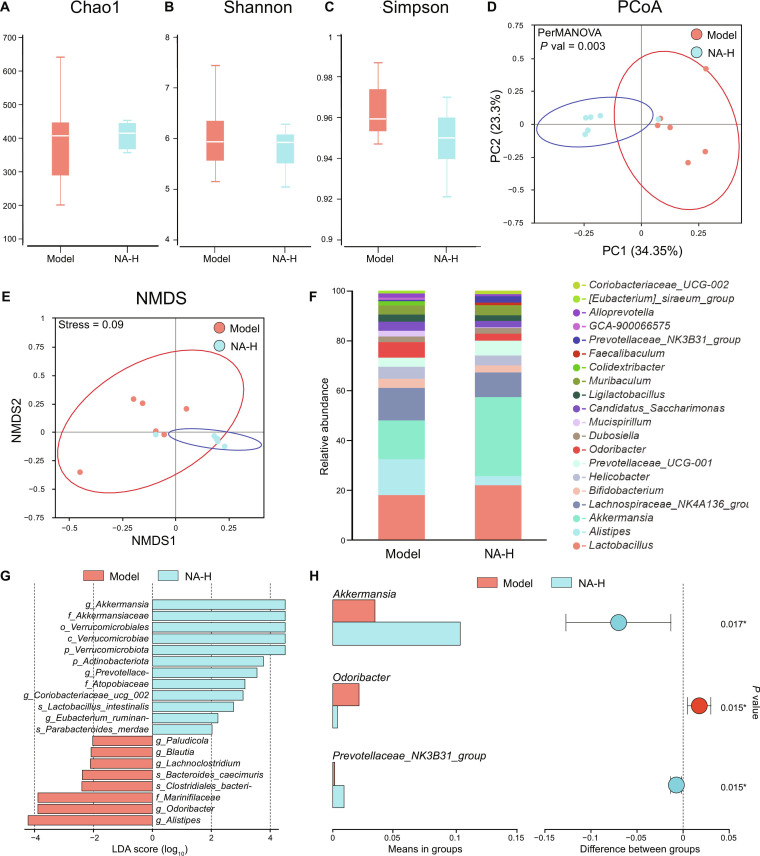
Nervonic acid (NA) supplementation reshapes gut microbiota. (A to C) α-diversity accessed by the Chao1, Shannon, and Simpson indices in the model and NA-H groups. (D and E) A plot of unconstrained principal coordinate analysis and nonmetric multidimensional scaling based on Bray–Curtis distances. (F) Relative abundance of the top 20 bacterial genera. (G) Linear discriminant analysis (LDA) effect size (LEfSe) analysis between the model and NA-H groups (LDA score ≥2). (H) The representative microbial community altered at the genus level between the model and NA-H groups. *n* = 6.

Growing clinical evidence reveals that PD is associated with gut microbiota dysbiosis, wherein dysregulated microbial communities may exacerbate the neurodegeneration in PD by secreting inflammatory cytokines or metabolites in turn [42]. This recognition has positioned gut microbiota remodeling as a promising therapeutic strategy for PD [43]. Given the marked intestinal retention of NA after oral administration, it is hypothesized that NA may possess potential microbiota-mediated neuroprotective effects. Therefore, the fecal samples from the model group and the NA-H group were analyzed through 16S rDNA sequencing to determine the changes in bacterial abundance after NA administration. The results show that the richness and uniformity of gut microbiota in PD mice were only slightly affected by NA treatment, as evidenced by minor differences in the Chao1, Shannon, and Simpson alpha diversity of the gut microbiota between the NA group and the model group (Fig. 6A to C). However, NA treatment induced marked beta diversity changes. Both principal coordinate analysis and nonmetric multidimensional scaling of amplicon sequence variants scored by Bray–Curtis dissimilarity showed that the gut microbiota’s structure and community composition in the NA group differed from that of the model group, with a distinct separation (Fig. 6D and E). Additionally, the permutational multivariate ANOVA test revealed a significant difference (*P* value = 0.003) in the bacterial community structure between these groups (Fig. 6D). Collectively, these results demonstrated that NA treatment reverses MPTP-induced alteration of the gut microbiota.

Next, the relative abundance of different bacterial taxa in different groups was characterized. Notably, NA treatment markedly increased the relative abundance of beneficial taxa, including *Lactobacillus* and *Akkermansia*, while reducing taxa, including *Alistipes*, *Odoribacter*, and *Mucispirillum* (Fig. 6F). The linear discriminant analysis effect size algorithm was then employed to perform linear discriminant analysis and identify microbial taxa that showed significant differences in abundance after NA treatment. At the genus level, the NA group exhibited a higher abundance of *Akkermansia*, *Prevotellaceae_NK3B31_group*, and *Lactobacillus*, while *Alistipes* and *Odoribacter* were abundant in the model group (Fig. 6G). Meanwhile, the *t* test also suggested that NA treatment reshaped the gut microbiota in PD mice, appearing as increased *Akkermansia* and *Prevotellaceae_NK3B31_group* and decreased *Odoribacter* (Fig. 6H). The enrichment of *Akkermansia* and *Prevotellaceae_NK3B31_group*, species known for protecting intestinal barriers and reducing systemic inflammation, suggests that NA may indirectly alleviate neuroinflammation via microbiota-derived metabolites like short-chain fatty acids (SCFAs) [44]. Moreover, *Odoribacter* is typically found to be elevated in PD and is also associated with neuroinflammation [45]. Notably, the results showed that NA treatment reversed the pathological increase in *Odoribacter*, potentially helping to mitigate neuroinflammation.

Together, the observed shift in beta diversity reflects a restructuring of the gut microbiota. While this structural change alone does not confirm functional recovery, the specific enrichment of SCFA-producing and anti-inflammatory genera, such as *Akkermansia* and *Prevotellaceae*, within the NA-treated group strongly suggests a functional transition toward a neuroprotective phenotype. This associative remodeling likely creates a favorable systemic environment that complements the central therapeutic effects of NA.

## Discussion

**Fig. 7.**
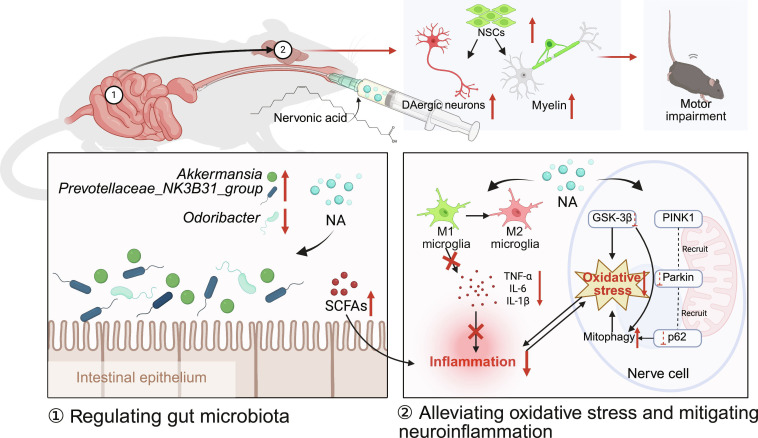
Schematic diagram illustrating the neuroprotective mechanism of nervonic acid (created in https://BioRender.com).

The present study has revealed the multidimensional neuroprotective effects of NA in mitigating the pathogenesis of PD, demonstrating its capacity to mitigate oxidative stress, relieve neuroinflammation, coordinate myelin regeneration, and modulate the gut microbiota. Our findings reveal that NA alleviates mitochondrial dysfunction by regulating PINK1–Parkin-mediated mitophagy, thus relieving oxidative stress. Meanwhile, NA promotes the polarization of microglia toward the M2 phenotype, thereby alleviating neuroinflammation. Additionally, NA enhances ceramide synthesis, thereby promoting sphingolipid metabolism, which is critical for maintaining neuronal membrane integrity and myelination. Notably, NA reshapes gut microbiota by increasing the abundance of anti-inflammatory taxa such as *Akkermansia* and *Prevotellaceae* while decreasing the abundance of pro-inflammatory taxa such as *Odoribacter*. These results highlight NA as a promising therapeutic candidate for PD, targeting both central and peripheral pathological mechanisms (Fig. 7).

This work extends previous studies on the neuroprotective role of NA by integrating the effects of NA on mitochondrial and gut–brain axis modulation. While previous research focused on NA’s ability to mitigate oxidative stress and improve motor symptoms [17,19], we present the first evidence that NA enhances mitophagy through the PINK1–Parkin signaling pathway, which is a key mechanism in PD progression. This finding aligns with emerging strategies targeting mitochondrial dysfunction in neurodegenerative diseases [8,46]. Additionally, we observed an up-regulation of ceramide species, highlighting NA’s involvement in sphingolipid metabolism, which is vital for oligodendrocyte function and myelin regeneration [33]. These results help connect NA’s biochemical properties to its therapeutic effects, especially for PD-associated white matter lesions.

Regarding the restoration of NeuN, nestin, and MBP, our findings suggest a synergistic dual mechanism involving both direct neuroprotection and immunomodulation. Directly, NA enhances mitophagy and suppresses oxidative stress to perform neuroprotection effect. Furthermore, as a biosynthetic precursor for sphingolipids, NA serves as a metabolic substrate for myelin repair. Indirectly, the NA-induced shift from neurotoxic M1 to neuroprotective M2 microglia mitigates the inflammatory environment, thereby creating a beneficial environment that secondarily promotes neuronal survival and oligodendrocyte differentiation.

Furthermore, the modulation of gut microbiota adds a novel dimension to the mechanism of NA. As *Akkermansia muciniphila* is virtually the only representative of *Akkermansia* in the gut, the increased *Akkermansia* on the genus level often represented the up-regulation of the species *A. muciniphila* [44]. *A. muciniphila* plays a key role in the enhancement of the integrity of the intestinal barrier, thereby regulating immunity and mitigating the neuroinflammation [44]. *Prevotellaceae_NK3B31_group* belongs to the family *Prevotellaceae*, which has been reported to be markedly reduced in PD [47]. Studies have demonstrated that the death of *Prevotellaceae* can cause the degeneration of SCFAs and lead to systemic inflammation [48]. SCFAs, particularly butyrate, have been suggested as key mediators in the microbiome–gut–brain axis, which can enhance myelin regeneration and attenuate neuroinflammation via direct humoral effects [49,50]. Our findings indicate that NA treatment may elevate the level of SCFAs by increasing the abundance of bacteria producing SCFAs, including *Akkermansia* and *Prevotellaceae*, suggesting a potential therapeutic effect on gut health, which in turn could influence neuroinflammatory processes.

Despite the advancements made in this study, several limitations must be considered. First, while NA demonstrated dose-dependent effects, the optimal therapeutic dosage and long-term safety profile have not been characterized. Second, regarding the specific mechanism of ceramide in neuroprotection, although our metabolomic data strongly imply that NA functions as a substrate to fuel the synthesis of protective long-chain ceramides, this study did not employ ceramide synthase inhibitors to block this metabolic flux. Therefore, while biochemical logic is reasonable, future studies using specific inhibitors are warranted to definitively demonstrate the necessity of ceramide up-regulation in NA’s neuroprotective effects. Third, although our results indicate that NA regulates gut microbiota and potentially elevates SCFA levels, we did not perform fecal microbiota transplantation or antibiotic depletion experiments to definitively isolate the direct contribution of the gut flora to neuroprotection. Therefore, the microbial remodeling observed here should be interpreted as an associative mechanism that complements the central effects of NA. Additionally, the precise molecular mechanisms by which microbial metabolites modulate neuroinflammation remain undefined. Future studies should utilize fecal microbiota transplantation models and direct metabolite profiling to establish the causal link gut-derived metabolites and NA-mediated neuroprotection.

In summary, this study established NA as a multidimensional neuroprotective agent to alleviate the core pathologies of PD through mitochondrial, anti-inflammatory, myelination, and gut microbial pathways, which distinguishes NA from traditional monotarget therapies. Although further research is required to address current limitations, our research provided a strong foundation for developing NA-based therapies, offering a novel strategy for PD treatment.

## Data Availability

The data that support the findings of this study are available from the corresponding authors upon reasonable request.
