# Supplementary material for: Nervonic Acid from Malania oleifera Reverses Parkinson’s Disease by Regulating Oxidative Stress, Neuroinflammation, and Gut Microbiota
Source: Biomater Res. 2026 May 8;30:0349. doi: 10.34133/bmr.0349 (PMC13153457; doi:10.34133/bmr.0349)
Supplement: Supplementary 1 — Figs. S1 to S6 [file bmr.0349.f1.docx]

**Supporting Information**

**Nervonic acid from Malania oleifera reverses Parkinson’s Disease by regulating oxidative stress, neuroinflammation and gut microbiota**

**
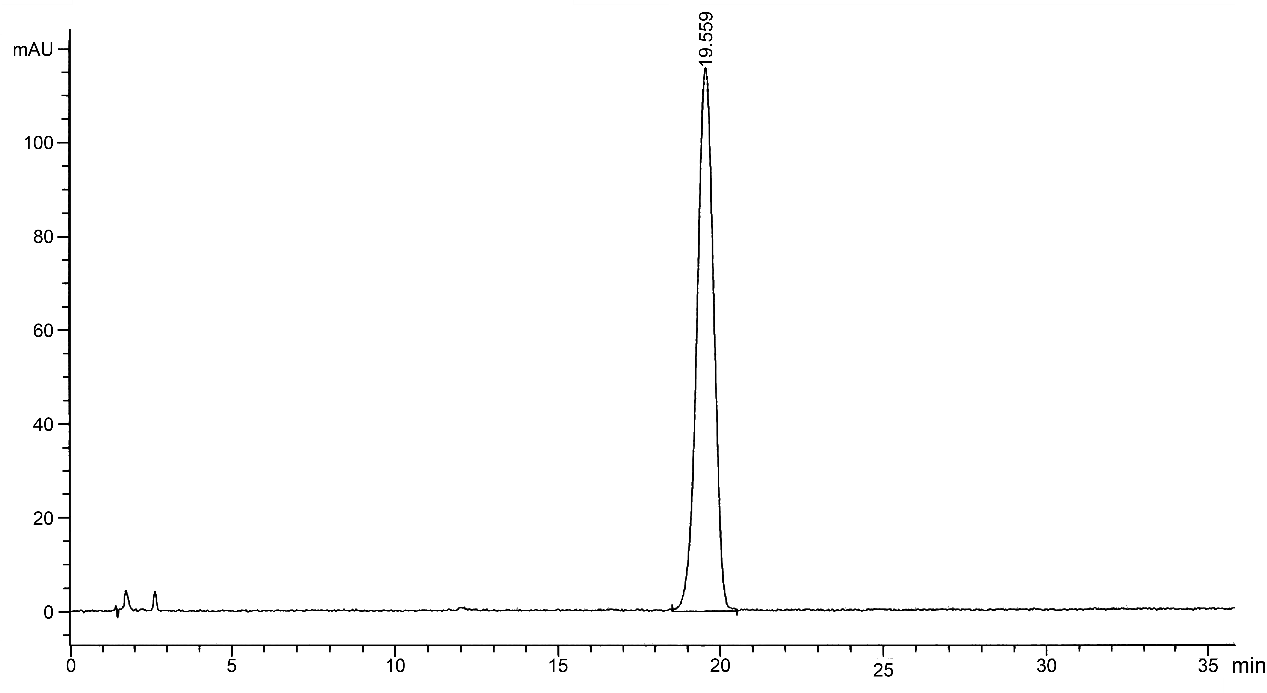
**

**Figure S1.** HPLC chromatogram of NA isolated from *Malania oleifera* (Retention time: 19.559 min).


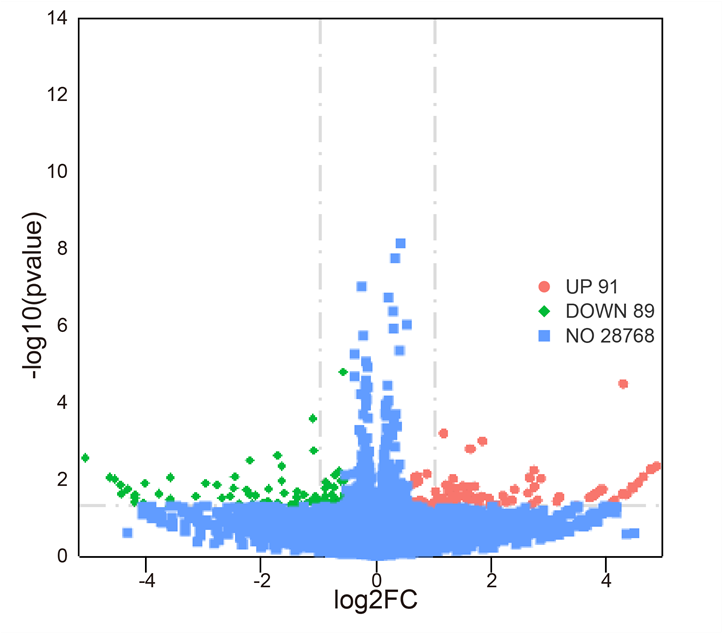


**Figure S2.** Volcano plot of the statistical difference of the DEGs.


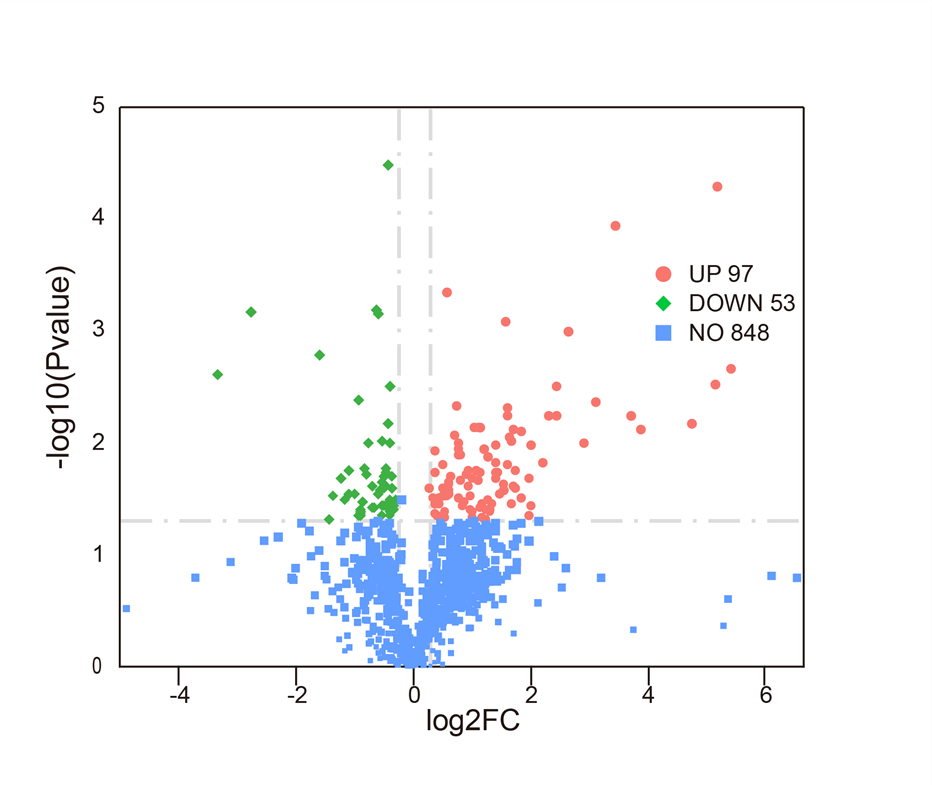


**Figure S3.** Volcano plot of the statistical difference of the DAMs. (|log2(FC)| ≥ 0.26, *p* < 0.05, VIP > 1)


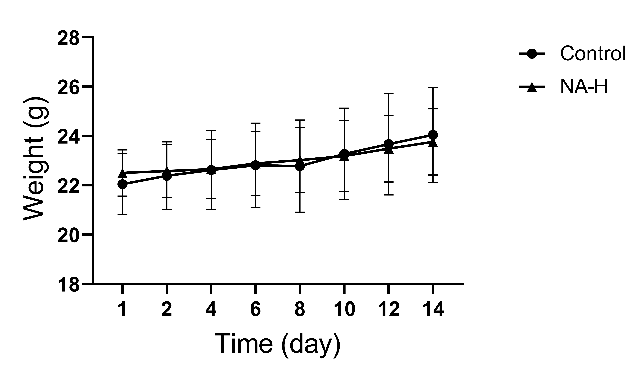


**Figure S4.** Body weight of the animals during NA administration..


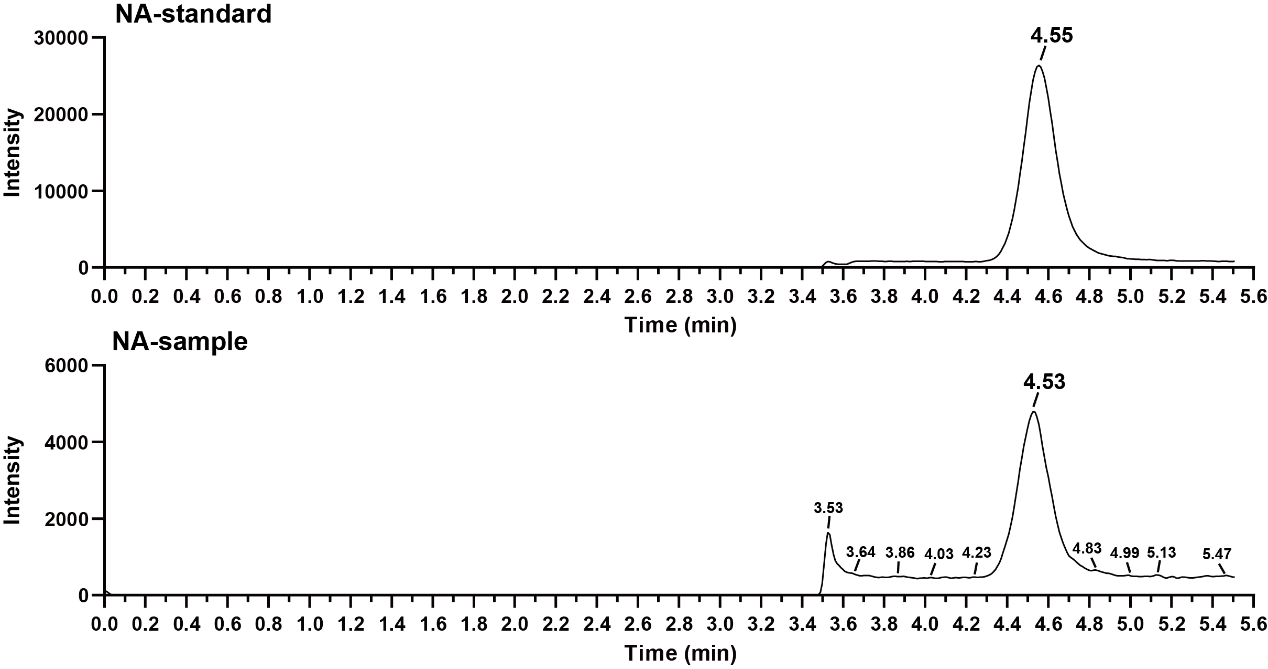


**Figure S5.** Representative HPLC chromatogram of NA standard sample and NA extracted from brain tissue.


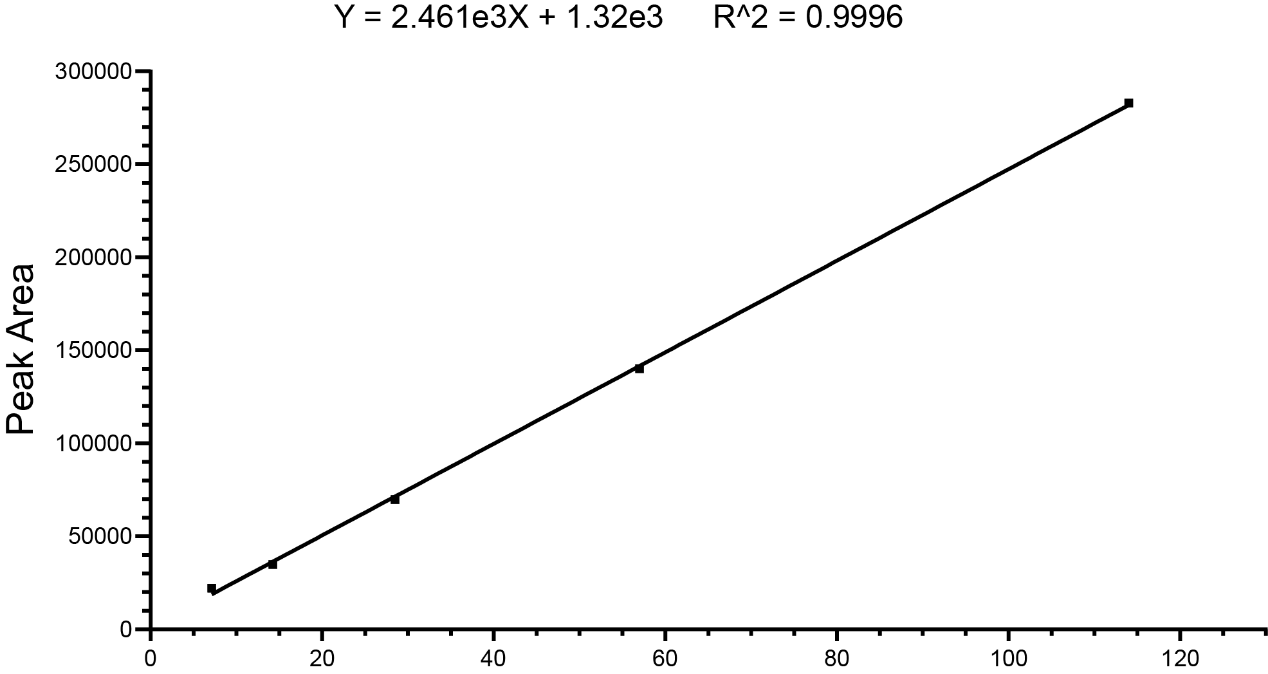


**Figure S6.** The standard curve of NA determined by HPLC-MS/MS
